# Acetaminophen Induces an Antioxidative Response in Lettuce Plants

**DOI:** 10.3390/plants10061152

**Published:** 2021-06-06

**Authors:** Inês Leitão, Luisa L. Martins, Luisa Carvalho, M. Conceição Oliveira, M. Matilde Marques, Miguel P. Mourato

**Affiliations:** 1 LEAF—Linking Landscape, Environment, Agriculture and Food, Instituto Superior de Agronomia, Universidade de Lisboa, 1349-017 Lisboa, Portugal; inesibleitao@isa.ulisboa.pt (I.L.); luisalouro@isa.ulisboa.pt (L.L.M.); lcarvalho@isa.ulisboa.pt (L.C.); 2 Centro de Química Estrutural, Instituto Superior Técnico, Universidade de Lisboa, 1049-001 Lisboa, Portugal; conceicao.oliveira@tecnico.ulisboa.pt (M.C.O.); matilde.marques@tecnico.ulisboa.pt (M.M.M.)

**Keywords:** acetaminophen, *Lactuca sativa*, emerging pollutants, pharmaceuticals, oxidative stress

## Abstract

Contaminants of environmental concern, like pharmaceuticals, are being detected in increasing amounts in soils and irrigation waters and can thus be taken up by plants. In this work, the uptake of acetaminophen (ACT) by lettuce plants was evaluated through a hydroponic experiment at different concentrations (0, 0.1, 1 and 5 mg L^−1^ ACT). The pathways related to oxidative stress induced by ACT were studied in lettuce leaves and roots at 1, 8 and 15 days after exposure. Stress indicators such as hydrogen peroxide and malondialdehyde (MDA) contents were analyzed, revealing increases in plants contaminated with ACT in comparison to control, confirming the occurrence of oxidative stress, with the exception of MDA in leaves. The enzymatic activities of catalase, superoxide dismutase, guaiacol peroxidase, ascorbate peroxidase and glutathione peroxidase, directly involved in the antioxidative system, showed significant differences when compared to control plants, and, depending on the enzyme and the tissue, different trends were observed. Glutathione reductase revealed a decrease in contaminated leaves, which may imply a specific impact of ACT in the glutathione cycle. Significant increases were found in the anthocyanin content of leaves, both with exposure time and ACT concentration, indicating an antioxidative response induced by ACT contamination.

## 1. Introduction

Plants can suffer different abiotic stresses, such as soil and water contamination by inorganic or organic products. Pharmaceuticals are documented to be present in surface and groundwater, manure and soil [1,2,3,4]. Despite the recognition that this group of contaminants may pose environmental concerns, there is still a lack of information on how plants cope with them.

The presence of pharmaceutical products in different environmental compartments results from different activities, including the incorrect disposal of drugs by humans, the non-efficient removal of drugs during wastewater treatments, and the use of a wide range of drugs in veterinary and livestock activity [5,6].

Since the presence of organic contaminants, like pharmaceutical products, is growing in different ecosystems, it is important to address how plants deal with this exposure. Previous studies reported that the uptake of pharmaceuticals by different plants could occur due to their ubiquitous presence in soils, irrigation water and wastewaters [7,8,9].

Depending on species, type of contaminant and growing conditions, plants develop distinct strategies to deal with stress conditions as a way to prevent or slow down the assimilation of contaminants and consequently avoid their excessive accumulation in tissues. Reactive oxygen species (ROS) occur naturally in cells; however, under stress conditions, the equilibrium is disturbed, causing oxidative stress [10]. As a result of plant-contaminant interaction, a disturbance in ROS content, such as hydrogen peroxide (H_2_O_2_), singlet oxygen, superoxide and the hydroxyl radical, may occur [11]. 

Most ROS are produced in mitochondria, chloroplasts, peroxisomes, the plasma membrane and the apoplast [11]. Particularly in the apoplast, different enzymes such as oxalate and amine oxidases are sources of H_2_O_2_. Some enzymes act directly on the production or scavenging of ROS, while others constitute a part of more complex systems, such as the ones involved in the ascorbate-glutathione cycle. For instance, superoxide dismutase (SOD) activity is on the frontline of defense mechanisms against oxidative stress, since its function is to catalyze the dismutation of superoxide radical to produce H_2_O_2_ and oxygen [12]. Catalase (CAT) function is related to the scavenging of H_2_O_2_ and is mostly present in peroxisomes. Despite its crucial action on ROS and its fast catalytic turnover rate, CAT affinity to the H_2_O_2_ molecule is lower compared to that of ascorbate peroxidase (APX) [13]. Peroxidases, including APX, guaiacol peroxidase (GPOD) and glutathione peroxidase (GPX), are also responsible for the degradation of H_2_O_2_ from cells [14]. GPOD, located in vacuoles and apoplasts, uses different phenolic substrates in the scavenging of ROS. The elimination of H_2_O_2_ through GPX activity occurs using reduced glutathione (GSH) as an electron donor, resulting in a molecule of oxidized glutathione (GSSG). GPX competes directly with CAT since it is responsible for H_2_O_2_ scavenging when low levels of ROS occur in cells. Moreover, GPX and APX increase their activity when CAT activity is inhibited [13,15]. Since GPX interferes with the ascorbate-glutathione pathway, it is important to assess the glutathione reductase (GR) enzyme as well. GR is responsible for the production of GSH resulting from the oxidation of NADPH [16]. The regeneration of GSH is one of the most important factors in plant antioxidant defense mechanisms [13]. According to Mhamdi et al. [17], the ascorbate-glutathione pathway may increase its activity in plants with lower CAT and GR levels, contributing to the elimination of ROS excess in plants with lower CAT and GR levels. 

Acetaminophen (ACT), also known as paracetamol, is one of the most widely used drugs in the world, being used in the treatment of fever and pain [18]. Fram and Belitz [4] analyzed 1231 groundwater samples used for drinking water where a maximum concentration of 1.89 μg L^−1^ of acetaminophen was found. Studies to assess acetaminophen uptake by different cultures have been reported with lupine (*Lupinus luteolus*), barley (*Hordeum vulgaris*), reed (*Phragmites australis*) and Indian mustard (*Brassica juncea* L. Czern.) [19,20]. 

In this work, we studied the effect of acetaminophen on *Lactuca sativa* L. plants. The choice of this pharmaceutical product was based on its significant consumption in Portugal, according to reports from INFARMED, the national authority for drugs and health products [21]. Adding to the current knowledge about the uptake of pharmaceuticals by plants, we intended to address further issues related to lettuce metabolism to understand how plants cope with low-concentration and short-term exposure to pharmaceuticals. These concentrations were selected in order to induce a measurable stress response but not high enough to cause the death of the plant and also because these would be the concentrations indicative of what has been found in the literature. Indeed, the range of concentrations in the literature is wide; for instance, Kotyza et al. [20] studied the effects of acetaminophen in *Armoratia rusticana* and *Linum usitatissimum* (both at 30 mg L^−1^) and *Hordeum vulgare, Lupinus luteolus* and *Phragmites australis* (all in a range of 15–181 mg L^−1^). A full characterization of the enzymatic antioxidant system was thus carried out in lettuce leaves and roots to clarify the main defense mechanisms used by lettuce plants against ACT-induced stress.

## 2. Results and Discussion

### 2.1. Acetaminophen Uptake in Leaves and Roots

Lettuce growing in hydroponics was contaminated with ACT (0.1, 1 and 5 mg L^−1^) added to the Hoagland solution. Afterwards, ACT was determined in lettuce leaves and roots through an LC-MS experiment. ACT can be taken up by plants, and, in Supplemental Figure S1, a typical chromatogram is presented (for roots and leaves of lettuce growing with 0.1 mg L^−1^ ACT, after 15 days of exposure) confirming the presence of ACT in contaminated lettuce plants.

**Figure 1:**
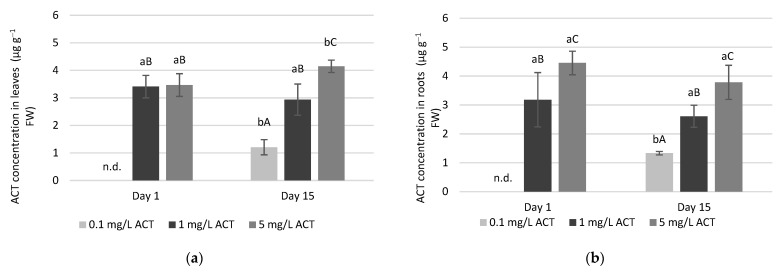
ACT concentration in leaves (**a**) and roots (**b**) in *Lactuca sativa* plants contaminated with ACT. n.d.: not detected (below the limit of quantification). Different uppercase letters represent significant differences between different ACT concentrations at the same timepoint, and different lowercase letters represent significant differences between treatment duration for the same ACT concentration.

As can be seen in Figure 1, on day 1, no ACT was detected (below the limit of quantification), neither in leaves nor in roots for the lower ACT concentration of 0.1 mg L^−1^. For longer periods of time and for the two highest concentrations, ACT was detected, at similar levels, both in leaves and in roots. For day 1, under 1 mg L^−1^ ACT exposure, the drug was detected in roots and leaves, at 3.18 ± 0.94 µg g^−1^ and 3.41 ±0.41 µg g^−1^, respectively. For the highest concentration of 5 mg L^−1^ ACT, the values of 4.45 ± 0.41 µg g^−1^ and 3.46 ± 0.41 µg g^−1^ were found in roots and leaves, respectively, on the same day. For each day and both in leaves and in roots, ACT contents increased with increasing ACT levels in the nutrient solution, with the exception of day 1 for leaves. Comparing day 1 with day 15, a significant increase in ACT concentration was only detected for the lowest ACT concentration of 0.1 mg L^−1^ and for leaves at the highest concentration of 5 mg L^−1^.

### 2.2. Biomass and Chlorophyll Parameters

The root and leaf mass were measured on each harvest day for the three ACT concentrations under study, but, despite the wide range of concentrations, from 0.1 to 5 mg L^−1^, no differences in these masses were detected in relation to control plants, and no visible damage was detected in the exposed plants (Supplemental Figure S2). Biomass parameters were previously documented by Favaro et al. [22], who found no differences in fresh weight (FW) after the exposure of lettuce to organic contaminants (biopesticides and deltamethrin). Although other authors found differences in terms of plant growth and foliar damage after the application of ACT [19], that was not the case in the present work, possibly because the concentrations used were lower (and more realistic) compared to this latter study on *Armoracia rusticana* L. plants. Our results show that, in the range of concentrations used, the effect of ACT in lettuce plants is not severe enough to affect plant growth and that lettuce has efficient defense mechanisms. Hence, it is important to clarify the metabolic effect and the main defense mechanisms activated by lettuce plants under ACT stress.

**Figure 2:**
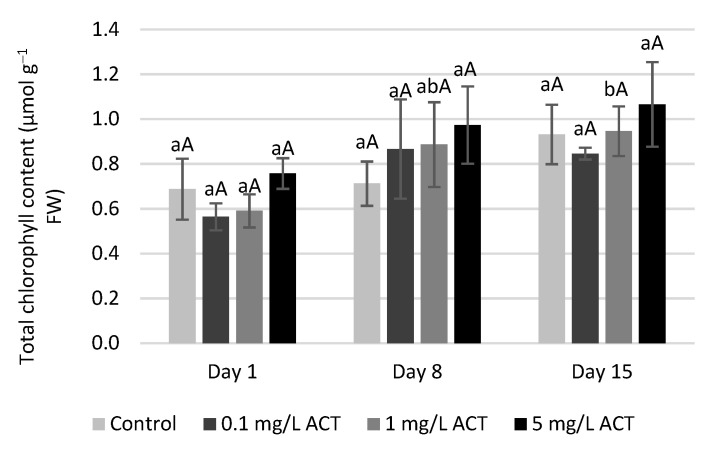
Total chlorophyll content (µmol g^−1^ FW) of *Lactuca sativa* contaminated with ACT. Different uppercase letters represent significant differences between different ACT concentrations at the same timepoint, and different lowercase letters represent significant differences between treatment duration for the same ACT concentration.

**Figure 3:**
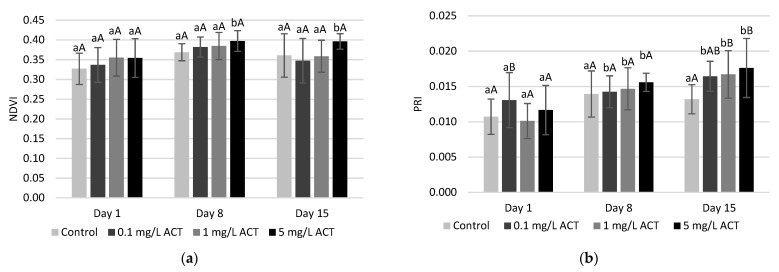
NDVI (**a**) and PRI (**b**) of *Lactuca sativa* contaminated with ACT. Different uppercase letters represent significant differences between different ACT concentrations at the same timepoint, and different lowercase letters represent significant differences between treatment duration for the same ACT concentration.

In Figure 2 and Figure 3, the effects of ACT on the photosynthetic system are presented. The total chlorophyll content (Figure 2) after different ACT treatments remained similar to that of the control, and, although some differences (mainly an increasing trend) were found, they were not statistically significant. Alkimin et al. [23] described some differences in chlorophyll *b* content for *Lemna minor* plants under acetaminophen contamination, even at lower concentrations (5 and 25 µg L^−1^) than those used in the current study, but they reported no differences in total chlorophyll content. By contrast, Hu et al. [24] reported an increase in total chlorophyll over time (30 and 45 days) in lettuce plants exposed to 1.5 mg L^−1^ of the endocrine disruptor bisphenol A.

The NDVI (normalized difference vegetation index) and PRI (photochemical reflectance index) were measured in situ (Figure 3a,b). NDVI is related to the plant’s photosynthetic capacity and photosynthetic rates. NDVI requires two wavelengths, one for the peak of visible light absorption by chlorophyll, while a second one will measure the infrared radiation reflected by the cellular structure of the leaves. For each ACT concentration, this parameter remained similar to that of control plants, which confirms that there were no changes in photosynthetic activity. PRI measurements show significant increases, both with time (8 and 15 days) and ACT concentrations. The PRI was originally developed to correlate changes in xanthophyll cycle pigments [25]. It stands as an indicator, not only of carotenoids/chlorophyll ratio but also for anthocyanins’ presence, since it compares the reflectance in the red and blue regions of the spectrum [26]. The PRI values obtained in the present work indicate that ACT contamination interferes with the pigment content and distribution and can reflect the overall variations in chlorophyll, carotenoid and anthocyanin contents.

### 2.3. Stress Indicators Analysis

**Figure 4:**
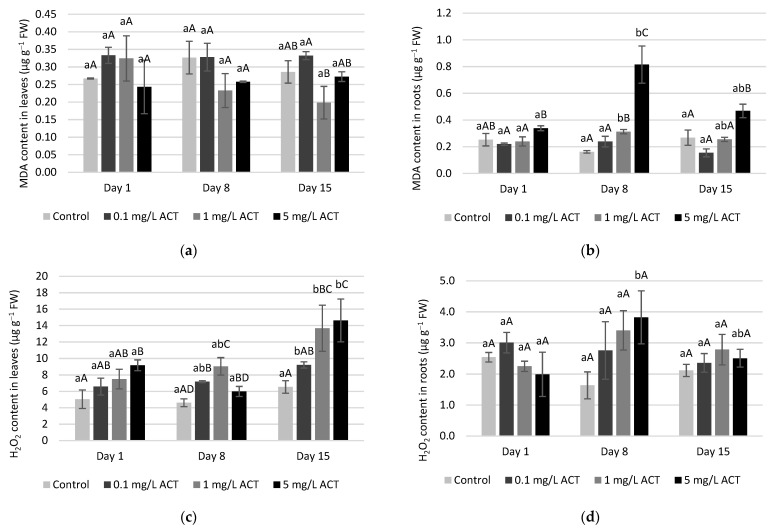
MDA and H_2_O_2_ contents (µg g^−1^ FW) in leaves (**a**,**c**) and roots (**b**,**d**) of *Lactuca sativa* contaminated with ACT. Different uppercase letters represent significant differences between different ACT concentrations at the same timepoint, and different lowercase letters represent significant differences between treatment duration for the same ACT concentration.

With respect to malondialdehyde (MDA) content, a measure of lipid peroxidation (as more lipid peroxidation leads to higher contents of MDA), significant increases were found in roots but not in leaves (Figure 4a,b). On days 8 and 15, roots showed higher contents of MDA when contaminated with 5 mg L^−1^ ACT. As the roots are immersed in the contaminated solutions, the lipid peroxidation effects are more noticeable in this plant part compared to the leaves, even though ACT was also detected in leaves, as seen in Figure 1. Several organic compounds have been reported to induce lipid peroxidation in lettuce plants [27]. 

**Figure 5:**
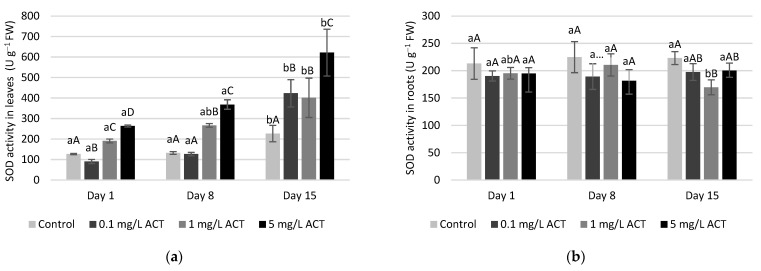
Enzymatic activity (U g^−1^ FW) of SOD in leaves (**a**) and roots (**b**) of *Lactuca sativa* contaminated with ACT. Different uppercase letters represent significant differences between different ACT concentrations at the same timepoint, and different lowercase letters represent significant differences between treatment duration for the same ACT concentration.

The presence of ACT also affected the H_2_O_2_ content (Figure 4c,d), and this was more evident in leaves compared to roots. It is possible to observe, in leaves, an increase in H_2_O_2_ content for the highest ACT concentration on the 1st day of exposure, for the concentrations of 0.1 and 1 mg L^−1^ ACT on day 8 and for 1 and 5 mg L^−1^ ACT on day 15. The increase in H_2_O_2_ content reflects an increase in ROS and is an indicator of oxidative conditions that can cause oxidative damage. This may also be linked to the measured increase of SOD activity in leaves (as presented below and shown in Figure 5), which shows significant increases for the same days and concentrations of ACT. The increase in SOD activity is also a possible indication of oxidative stress and intracellular increase of ROS.

### 2.4. Antioxidative Enzymes and Metabolites Related to Stress Response

**Figure 6:**
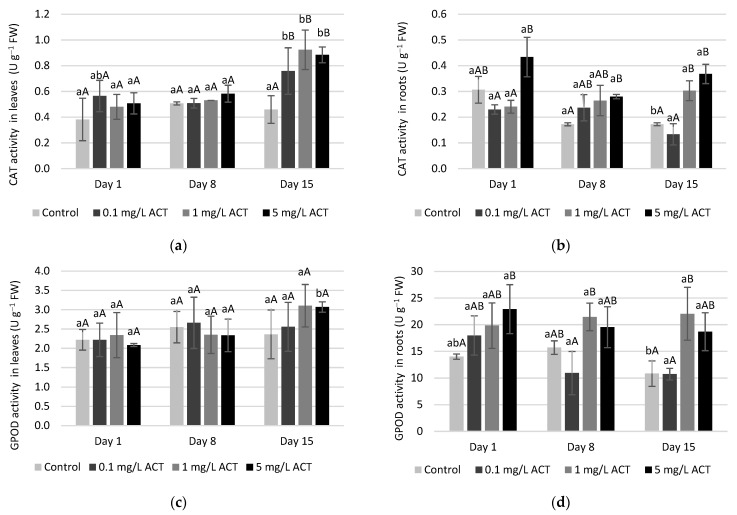
Enzymatic activity (U g^−1^ FW) of CAT in leaves (**a**) and roots (**b**) and GPOD in leaves (**c**) and roots (**d**) of *Lactuca sativa* contaminated with ACT. Different uppercase letters represent significant differences between different ACT concentrations at the same timepoint, and different lowercase letters represent significant differences between treatment duration for the same ACT concentration.

**Figure 7:**
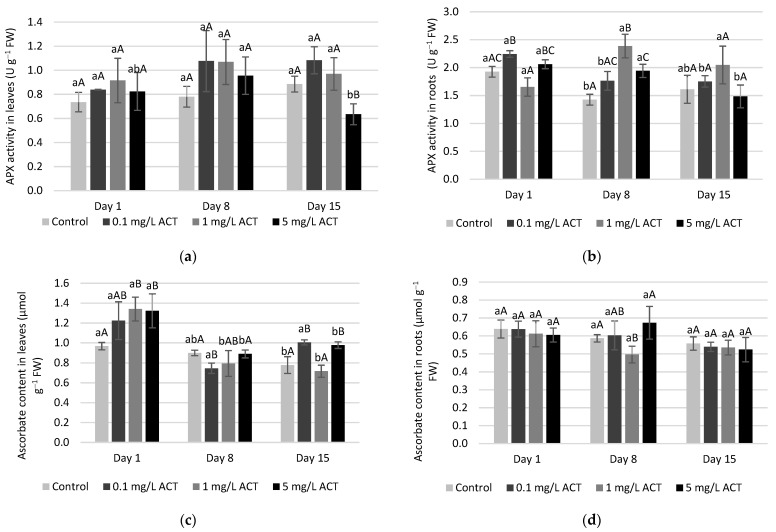
Enzymatic activity (U g^−1^ FW) of APX in leaves (**a**) and roots (**b**) and ascorbate content (µmol g^−1^ FW) in leaves (**c**) and roots (**d**) of *Lactuca sativa* contaminated with ACT. Different uppercase letters represent significant differences between different ACT concentrations at the same timepoint, and different lowercase letters represent significant differences between treatment duration for the same ACT concentration.

**Figure 8:**
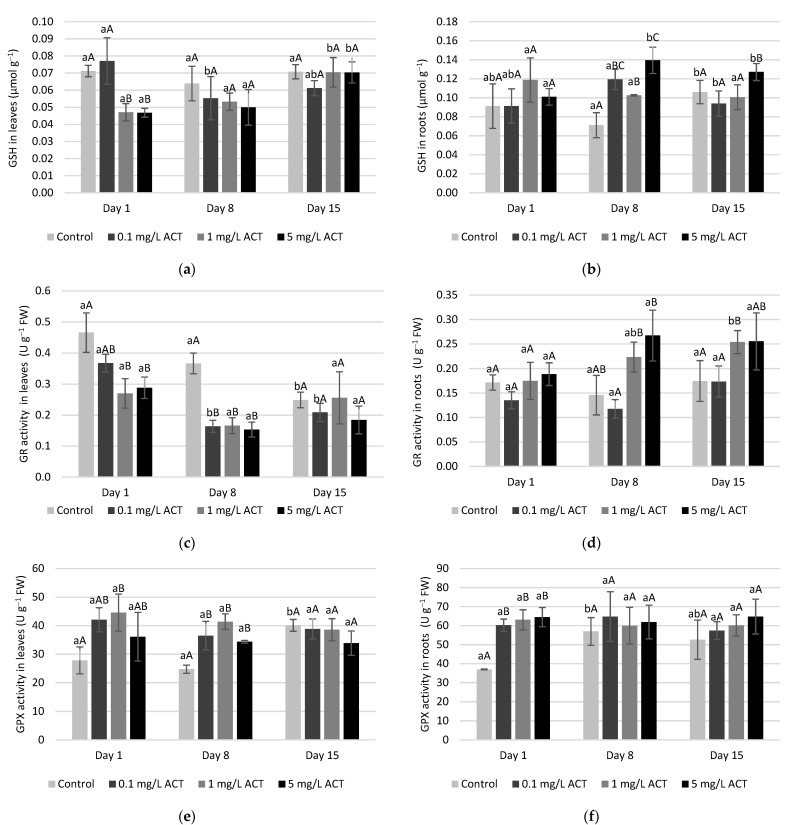
GSH content (µmol g^−1^ FW) in leaves (**a**) and roots (**b**) and enzymatic activities (U g^−1^ FW) of GR in leaves (**c**) and roots (**d**) and of GPX in leaves (**e**) and roots (**f**) of *Lactuca sativa* contaminated with ACT. Different uppercase letters represent significant differences between different ACT concentrations at the same timepoint, and different lowercase letters represent significant differences between treatment duration for the same ACT concentration.

The activities of SOD, CAT, GPOD, APX, GPX and GR were measured both in roots and in leaves for the 3 ACT concentrations and are presented in Figure 5, Figure 6, Figure 7 and Figure 8. 

The increase in SOD activity is a frequently triggered response to abiotic stress [28]. In the present work, we found that SOD activity in lettuce plants exposed to ACT is not as pronounced in roots as in leaves (Figure 5), suggesting that the presence of the superoxide radical (O_2_^•-^) is not as prominent in the roots. In fact, SOD does not seem to be induced by oxidative stress in the roots, as its activity did not increase with ACT compared to control. On the other hand, the increase in SOD activity in leaves is correlated with ACT exposure (with both time and ACT concentration) and might reflect an increase of superoxide radical content. Superoxide is a common ROS in non-stressed cells, but its presence may increase under biotic or abiotic stresses. Since SOD is present in different organelles, the content of H_2_O_2_ found in leaves might be a result of SOD activation in different compartments of the cells, such as chloroplasts and mitochondria [29,30].

Depending on in which organelle H_2_O_2_ production occurs, its scavenging may be performed by APX (in the chloroplasts) or by GPX and CAT if it occurs in cytosol or peroxisomes, respectively [13]. In the present work, CAT activity (Figure 6a,b) shows significant increases compared to control plants. For instance, an increase on day 15 for 1 and 5 mg L^−1^ ACT was found in both leaves and roots. In the case of leaves, this result can be explained by the high content of H_2_O_2_ mostly related to mitochondria and chloroplasts. This increase in CAT activity probably explains the fact that MDA in leaves did not significantly increase, showing that major oxidative damage was avoided. In roots, it is possible to observe that the H_2_O_2_ content was lower on day 15 when compared to day 8 and that result could be related to mitochondria. Thus, an increase in CAT activity may be the pathway of H_2_O_2_ reduction with a longer period of exposure, which suggests a good antioxidant response to ROS in root cells. An increase (days 8 and 15 for 1 mg L^−1^) in GPOD activity was also observed for the same tissue (Figure 6c,d). In fact, both CAT and GPOD act on H_2_O_2_ removal, albeit using different substrates. In the case of GPOD, an aromatic compound is used as electron donor to trigger the reaction of H_2_O_2_ elimination [31]. GPOD activity is also related to cell-wall properties since this enzyme is linked to cell-wall lignification; therefore, the increase in GPOD activity may confer better stability to root cells [32]. Indeed, as no visible damage was observed in leaves and roots during the exposure to ACT this may indicate efficient cell lignification, which may be linked to GPOD activity. This result will then explain why lipid peroxidation did not cause visible damage in roots.

As stated before, APX also takes part in H_2_O_2_ scavenging. During the exposure of lettuce to ACT, some significant increases were found for APX on day 8 in roots (Figure 7b). A concomitant decrease in ascorbate (AsA) contents was detected under the same conditions, probably related to the use of AsA by the enzyme. These results point to possible involvement in the response to oxidative stress [33]. However, APX activity did not play a major role in H_2_O_2_ removal as clear as the one observed for CAT and GPOD (Figure 6). In Figure 7c,d the results for AsA content are also presented, as AsA is an important antioxidant and is a substrate of APX (total ascorbate values are also presented as Supplemental Figure S3). In detail, for day 1, a significant increase was found in AsA content in leaves in comparison to control. In roots, a decrease of AsA content is observed for day 8 at 1 mg L^−1^ ACT concomitant with an intensification in APX activity for the same concentration on day 8. In general, APX and AsA contents were well correlated, since an increase in APX activity is frequently associated with a decrease in AsA contents, for instance like what was observed on day 8 in leaves, although most of the differences are not significant. Ascorbate is involved in several reactions and is considered one of the most significant species controlling ROS contents in cells, together with GSH [34]. Nevertheless, in this work, APX activity and ascorbate content did not vary much. This observation may be explained by the fact that AsA pools are less affected than GSH contents in some plants [35]. 

GSH content in leaves displayed some oscillations between days and ACT concentrations (Figure 8a,b; total glutathione values, and GSH/GSSG ratios are also presented in Supplemental Figure S3). However, it is possible to say that GSH levels in leaves of contaminated plants tended to be lower than in control plants. According to Noctor et al. [34], GSH status is considered a good indicator when the cellular redox state is changed, in particular when the content of ROS, such as H_2_O_2_, increases. The ascorbate-glutathione cycle is one of the most important pathways to regulate the redox state of cells. The linking between these two metabolites is then complemented by the enzymes APX and GR. GR activity results (Figure 8c,d) revealed a relation with the differences in GSH content, meaning that when GR activity decreases, it is possible to observe a decrease in GSH levels. This behavior is mostly observed in leaves, as the activity of GR was lower in contaminated leaves when compared to control. With different types of contaminants, similar behavior in lettuce, under arsenate and arsenite exposure, has been documented [36]. In fact, GSH biosynthesis may be triggered by different pathways and, depending on the type of stress, the accumulation of this metabolite may occur under oxidative stress conditions [37].

The lower GSH contents may be explained by the lower activity (or lower content) of GR during ACT exposure or by its use as a substrate of GPX activity in ROS scavenging processes. In line with the GR and GSH results, APX and AsA measurements indicate that the APX activity was increased on day 8 by 0.1, 1 and 5 mg L^−1^ ACT in leaves, reducing the ascorbate content and producing more dehydroascorbate, which will serve as the substrate for the reaction mediated by dehydroascorbate reductase (DHAR). Yet this slight increase in APX activity is not significant, being only significant for the AsA content at day 8 in 0.1 and 1 mg L^−1^ contaminations. At the same time, DHAR mediates the oxidation of GSH to GSSG, which will be the substrate for GR [38]. In leaves, this conversion is limited by the low GR activity, but in roots, the GSH recycling showed a better performance. In roots, both the GSH contents and the GR activity increased with ACT contamination levels and time of exposure. For instance, on day 8 significant increases were detected in GSH, while GR activity increased but not significantly. Therefore, dissimilar behavior between cells from roots and leaves is observed.

GPX also intervenes in GSH/GSSG conversion. The results (Figure 8e,f) for its activity show more significant increases for days 1 and 8 in leaves and only on day 1 in roots. Although GPX activity is known to be related with H_2_O_2_ scavenging [39], the link between H_2_O_2_ content and GPX activity was not very clear in the lettuce plants. This may indicate that GPX is not extensively involved in ROS removal, probably because GSH participates in other oxidative defense-related reactions. It is possible to observe that for day 15, GPX activity remained similar to control levels, albeit the H_2_O_2_ concentration in leaves increased in this period. This observation leads to the conclusion that GPX does not play a relevant role in H_2_O_2_ scavenging, while CAT (and GPOD to a lesser degree) is the enzyme that mostly increased its activity at day 15 in the leaves, probably as a response to the increase in H_2_O_2_ concentrations. As ROS contents must be kept under control, in order to reduce the negative impact on cells, the equilibrium between ROS scavenging enzymes is essential. The high content of H_2_O_2_ will keep some enzymes (CAT, SOD, GPOD) more active in ROS removal when compared to others (APX, GPX) [40].

**Figure 9:**
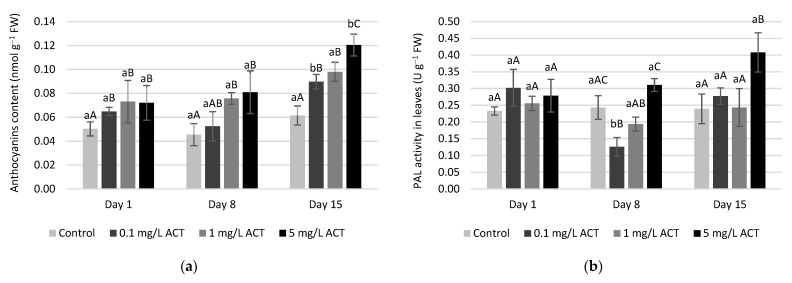
Anthocyanins content in leaves (**a**) and PAL activity (**b**) (U g^−1^ FW) of *Lactuca sativa* contaminated with ACT. Different uppercase letters represent significant differences between different ACT concentrations at the same timepoint, and different lowercase letters represent significant differences between treatment duration for the same ACT concentration.

Anthocyanin content (Figure 9) was also measured in leaves, and significant increases were found on days 8 and 15, with major increases in leaves contaminated with 1 and 5 mg L^−1^ of ACT. This was also observed by other authors, confirming that, under abiotic stress, anthocyanin content tends to increase [12,41,42]. In the specific case of ACT contamination, Alkimin et al. [23] reported some significant decreases in *Lemna gibba* species; however, applied concentrations were lower when compared to the ones studied in this work. Another consequence of anthocyanin increase is the possibility of binding with GSH forming complexes [43]. This may influence the availability of GSH to participate in the ascorbate-glutathione cycle and contribute to the lower concentrations of this metabolite in leaves under ACT contamination. 

**Figure 10:**
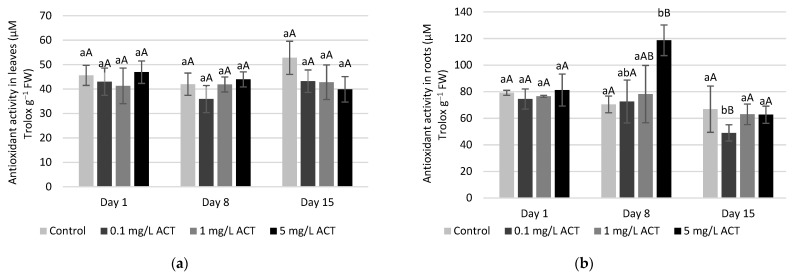
Antioxidant activity (μM Trolox g^−1^ FW) in leaves (**a**) and roots (**b**) of *Lactuca sativa* contaminated with ACT. Different uppercase letters represent significant differences between different ACT concentrations at the same timepoint, and different lowercase letters represent significant differences between treatment duration for the same ACT concentration.

The production of anthocyanins is directly related with phenylalanine ammonia lyase (PAL) activity (Figure 9b). This is a key enzyme of the phenylpropanoids pathway, which will be crucial to the production of anthocyanins. The treatment-related increased levels of anthocyanins match a higher activity of PAL. Significant increases compared to the control were observed on leaves after 15 days of exposure to 5 mg L^−1^ ACT. Nonetheless, the global level of antioxidant capacity in leaves and roots (Figure 10) remained statistically similar to control, with the exception of day 8 in roots under 5 mg L^−1^ ACT. The fact that no changes in overall antioxidant capacity occur may be explained by a good regulation of defense mechanisms and interactions between the metabolites and enzymes that constitute the response to induced oxidative stress.

## 3. Materials and Methods

### 3.1. Experimental Setup

Seeds of *Lactuca sativa* L. “Maravilha das Quatro Estações” were germinated in a substrate, where they were kept for 15 days until the development of the first leaf. The lettuce plants were then placed in a hydroponic Hoagland solution and developed in this medium for 21 days. On the 36th day of growth, plants were separated and transferred to pots containing different concentrations of ACT in Hoagland solution—0 mg L^−1^ (control), 0.1 mg L^−1^, 1 mg L^−1^ and 5 mg L^−1^. The contaminated solutions were changed every 5 days. All the treatments were performed in triplicate. During the experiment, the plants were placed in a controlled environment with temperatures between 20 °C and 25 °C, relative humidity of 60–65% with a 12-h light–dark cycle. On days 1, 8 and 15 after exposure to ACT, the lettuce plants were harvested. Biomass parameters, such as plant mass, the number of leaves and leaf and root mass, were measured.

### 3.2. Activity of Antioxidant Enzymes

The activities of CAT, GPOD, SOD, GPX, GR and APX were determined as previously described by Martins et al. [44] and Pinto et al. [45]. Regarding the PAL enzyme, approximately 500 mg of fresh material was crushed with inert sand in a mixture of 3 mM dithiothreitol (DTT) and 25 mM of sodium borate buffer (pH 8.8). Samples were centrifuged at 20,000× *g* (Sigma 03–18 K) for 20 min at 4 °C. Afterwards, samples were placed in tubes, and the reaction medium was composed of 5 mM of L- phenylalanine and 25 mM of sodium borate buffer (pH 8.8). A blank assay was performed in parallel. The samples were incubated for 2 h at 40 °C. To stop the reaction, a solution of 5 M HCl was added to each sample. Enzyme activity was expressed in U g^−1^ FW. All determinations were performed in triplicate (from three different plants).

### 3.3. Photosynthetic Parameters, Total Chlorophyll and Anthocyanin Content

Photosynthetic parameters, such as PRI (PlantPen PRI 300) and NDVI (PlantPen NDVI 200), were measured during the assay. A total of 10 leaves were selected, and 4 measurements for each leaf were obtained for each treatment. Results are expressed in arbitrary units. 

Total chlorophyll content was analyzed through a destructive technique, according to the method described by Carvalho et al. [46]. 

For anthocyanin content, 60 mg foliar disks were crushed in a cold mortar with 10 mL of methanol:HCl (90:10, *v*:*v*). The absorbance was determined according to the method described by Murray and Hackett [47]. Absorbance values (Analytic Jena SPECORD 200) for chlorophyll and anthocyanins were applied to the equations described by Sims and Gamon [26].

### 3.4. Hydrogen Peroxide Content and Lipid Peroxidation

The methodologies for H_2_O_2_ and MDA, as a measure of lipid peroxidation) extraction and quantification followed the protocol described by Fernandez et al. [48], using molecular absorption spectrophotometry (Analytic Jena SPECORD 200) with a calibration curve executed using the same conditions as the samples.

### 3.5. Glutathione and Ascorbate Content

The determination of glutathione was based on the method described in Singh et al. [49]. Samples were prepared from fresh material (500 mg), which was macerated with 6% metaphosphoric acid (2.5 mL) containing 1 mM EDTA, followed by centrifugation for 15 min at 22,000× *g* and 4 °C (Sigma 03–18 K). For reduced GSH determination, two separate measurements of total glutathione (TGSH) and oxidized glutathione (GSSG), levels were performed, using a Biotek Sinergy HT plate reader. GSH was calculated by subtracting the amount of GSSG from the total glutathione.

For AsA determination, 500 mg of fresh material was mixed with 5 mL of 100 mM Tris-HCl buffer containing 1.5 mM DTT and 1 mM EDTA, pH 7.5. The whole extraction procedure was conducted at 0–4 °C. After centrifugation (15 min at 22,000× *g* and 4 °C (Sigma 03–18 K)), the supernatant was collected. To determine reduced ascorbate, 200 μL of supernatant were added with 500 μL 150 mM KH_2_PO_4_, 5 mM EDTA solution and 100 μL of deionized water and stirred. After stirring for 10 min at room temperature, 100 μL of deionized water were added. To complete the color development, 400 μL of 10% thrichloroacetic acid, 400 μL of 44% orthophosphoric acid, 400 μL of 0.26 M 2,2′- bipyridyl in 70% ethanol and 200 μL of 11 mM FeCl_3_ were added to the mixtures. The mixture was stirred and left at 40 °C for 1 h. Absorbance readings were then performed in an Analytic Jena SPECORD 200 spectrophotometer.

### 3.6. Antioxidant Activity

For the measurement of antioxidant activity, 500 mg of fresh material was macerated in methanol. Antioxidant activity determination followed the method described by Brand-Williams et al. [50], and the results were expressed in μM Trolox g^−1^ FW.

### 3.7. Acetaminophen Identification and Quantification

To measure the uptake of ACT, frozen leaf and root tissues (≈500 mg) were crushed in a mortar with 1.5 mL of methanol. The samples were centrifuged at 20,000× *g* for 20 min at 4 °C, and the supernatant was collected and kept at 4 °C until further analysis. 

The identification and quantification of ACT in plant extracts was performed in an LC-MS system composed by an Elute UHPLC system and tandem Impact II QTOF mass spectrometer (Bruker Daltonics, Bremen, Germany) with an electrospray ion source (ESI). Chromatographic separation was carried out on a Luna C18 reversed-phase column 100 Å (150 × 3 mm, 3 µm particle size; Phenomenex). The mobile phase consisted of 0.1% (*v/v*) formic acid in water (A) and in acetonitrile (B). The elution gradient was as follows: 0–0.5 min, 5% B; 0.5–2 min, 5% B; 2–7 min, 40% B; 7–11 min, 95% B; 11–11.5 min, 95% B; 11.5–15 min, 5% B. The injected volume was 10 μL. The flow rate was 300 μL min^−1^, and the temperatures of the column and autosampler were maintained at 40 °C and 8 °C, respectively. The mass spectrometer was operated in the ESI positive mode. Sodium formate (10 mM) was used as internal calibrant before each analysis. Acquisitions were performed in full scan mode in the *m/z* 70 –1000 mass range, at a scan rate of 1 Hz. Data acquisition and processing were performed using Data Analysis 4.2, Target Analysis 1.3 and TASQ 1.4 software (Bruker Daltonics).

ACT was identified based on accurate *m/z* values released as protonated molecules [M+H]^+^, taking into account the accuracy and precision of the measurement parameters, such as error (ppm) and mSigma. As an internal standard, a solution of acetaminophen-D4, (100 µg mL^−1^) was used, to correct for experimental variability during sample preparation and analysis.

The estimation of the ACT concentrations was performed based on an external standard curve of acetaminophen (CAS: 103-90-2, Sigma Aldrich) prepared in the concentration range of 10 to 3 × 10^3^ ppb in methanol, similar to the medium used for plant analysis. The R^2^ for the regression data for ACT was always higher than 0.98, for all concentrations studied. The limit of detection (LoD) was 0.0042 µg g^−1^ FW, and the limit of quantification (LoQ) was 0.0013 µg g^−1^ FW (an average mass of 0.75 g was used in these calculations).

### 3.8. Statistical Analysis

All the analytical determinations were performed in triplicate. To assess significant differences between the different experimental conditions, the results were subjected to one-way ANOVA using the Tukey test to check for significant differences between means (P < 0.05). In the figures, error bars represent mean ± one standard deviation, different uppercase letters represent significant differences between different ACT concentrations at the same time point, and different lowercase letters represent significant differences between treatment duration for the same ACT concentration.

## 4. Conclusions

ACT uptake was detected in roots and leaves even for the lower concentrations of this contaminant (albeit below the limit of quantification), confirming that this class of pharmaceuticals may reach plants and constitutes a potential contamination hazard in edible vegetables. The fact that no loss of biomass or visual effects were detected in the plant make it particularly concerning, as it maintained a healthy look and might lead to its consumption.

ACT presence affected the normal oxidative status of lettuce plants. The results of the overall antioxidant response confirm that ACT induced oxidative stress and the plant responded by undergoing metabolic changes that ensured tolerance to phytotoxicity. In roots, CAT and GPOD were the most affected enzymes, and their activity increased for longer periods of exposure to ACT, inducing lower contents of H_2_O_2_, in order to replace the ROS equilibrium. However, in leaves, CAT revealed more significant increases than GPOD when compared to control. SOD activity in leaves was more affected than in roots, revealing significant increases at each timepoint for concentrations of 1 and 5 mg L^−1^ ACT. In roots the main differences caused by stress were found in the glutathione cycle, with an increase in GR activity and a consequent increase of GSH pools for days 8 and 15 of exposure to ACT. These changes may be related with possible detoxification processes involving GSH pathways.

## Data Availability

Data sharing is not applicable to this article.

## Supplementary Materials

The following are available online at https://www.mdpi.com/article/10.3390/plants10061152/s1, Figure S1: Typical chromatograms of ACT detected in lettuce leaves and roots, Figure S2: Lettuce plants on day 15 of contamination with ACT in different concentrations. Figure S3: Total Ascorbate and GSH content and GSH/GSSG ratio in leaves and roots of *Lactuca sativa* under contamination with ACT for up to 15 days.
