# Supplementary material for: Acetaminophen Induces an Antioxidative Response in Lettuce Plants
Source: Plants (Basel). 2021 Jun 6;10(6):1152. doi: 10.3390/plants10061152 (PMC8229777; doi:10.3390/plants10061152)
Supplement: Supplementary file 1 [file plants-10-01152-s001.zip › sup/FIGURE S1.pdf]

(a)

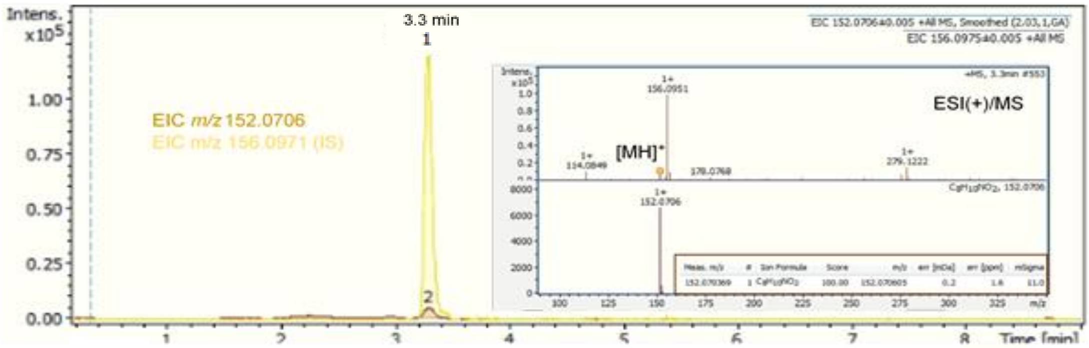

(b)

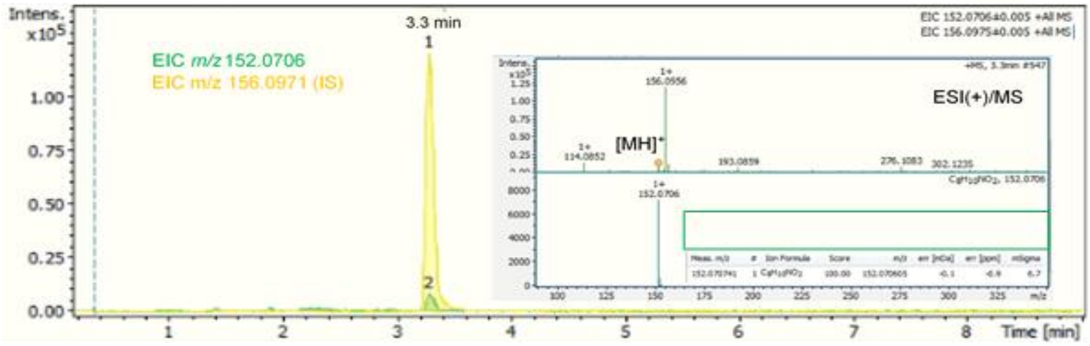

FIGURE S1 – Typical extracted ion chromatograms obtained in the ESI positive mode for the protonated molecules of ACT (m/z 152.0706) identified: (a) in roots and (b) in leaves, of the extracts of lettuce growing with 0.1 mg L^{-1} ACT, after 15 days of exposure. Peak 1 is due to the protonated molecule m/z 156.0975 of the internal standard ACT-D4.
