# Supplementary material for: Acetaminophen Induces an Antioxidative Response in Lettuce Plants
Source: Plants (Basel). 2021 Jun 6;10(6):1152. doi: 10.3390/plants10061152 (PMC8229777; doi:10.3390/plants10061152)
Supplement: Supplementary file 1 [file plants-10-01152-s001.zip › sup/FIGURE S2.pdf]

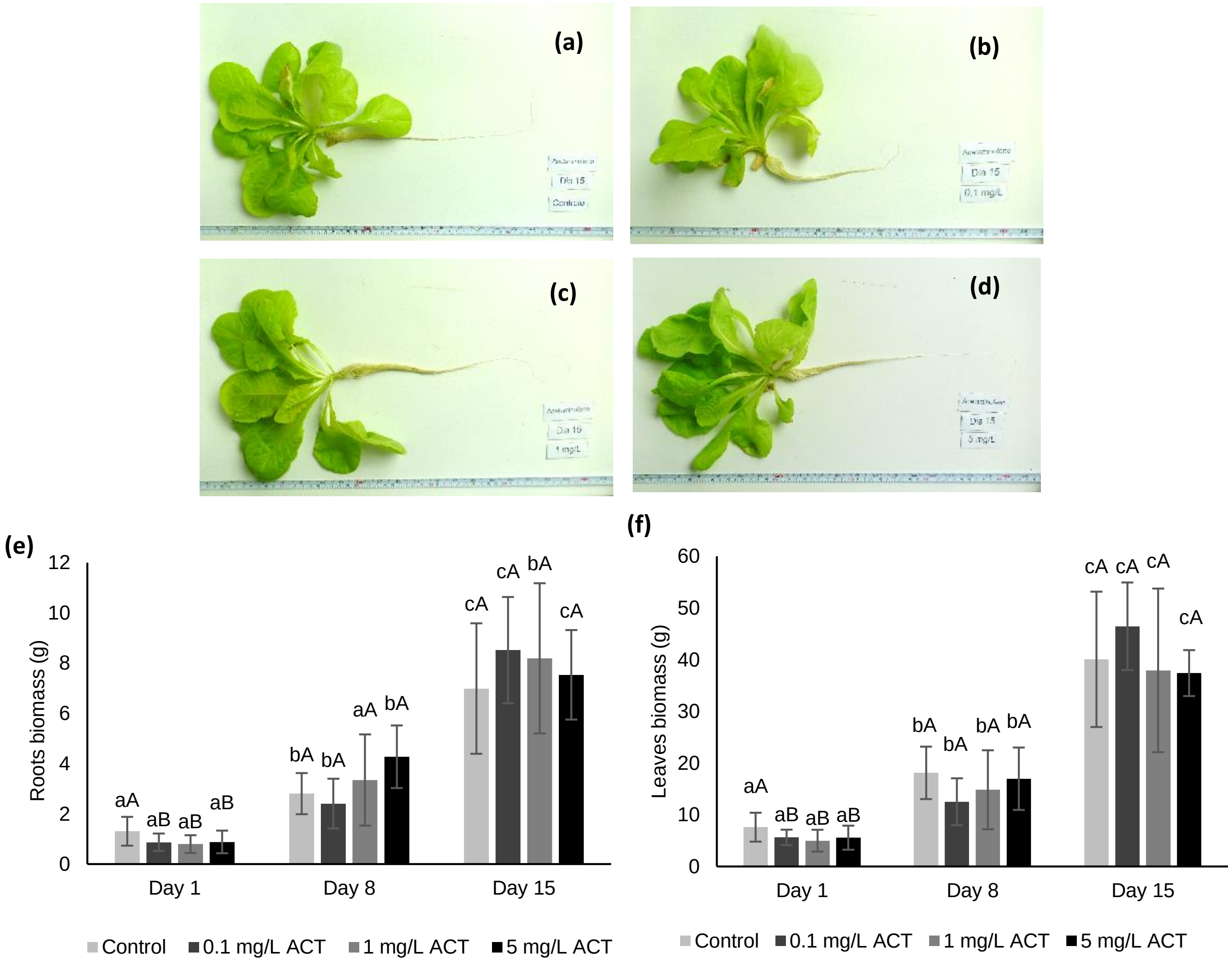

Figure S2 – Lettuce plants on day 15 of contamination with ACT in different concentrations. Control (a), 0.1 mg L^{-1} (b), 1 mg L^{-1} (c), 5 mg L^{-1} (d) and root (e) and leaf (f) masses. Different uppercase letters represent significant differences between different ACT concentrations at the same timepoint and different lowercase letters represent significant differences be-tween treatment duration for the same ACT concentration.
