# Supplementary material for: Acetaminophen Induces an Antioxidative Response in Lettuce Plants
Source: Plants (Basel). 2021 Jun 6;10(6):1152. doi: 10.3390/plants10061152 (PMC8229777; doi:10.3390/plants10061152)
Supplement: Supplementary file 1 [file plants-10-01152-s001.zip › sup/Figure S3_Asc GSH Total_V2.pdf]

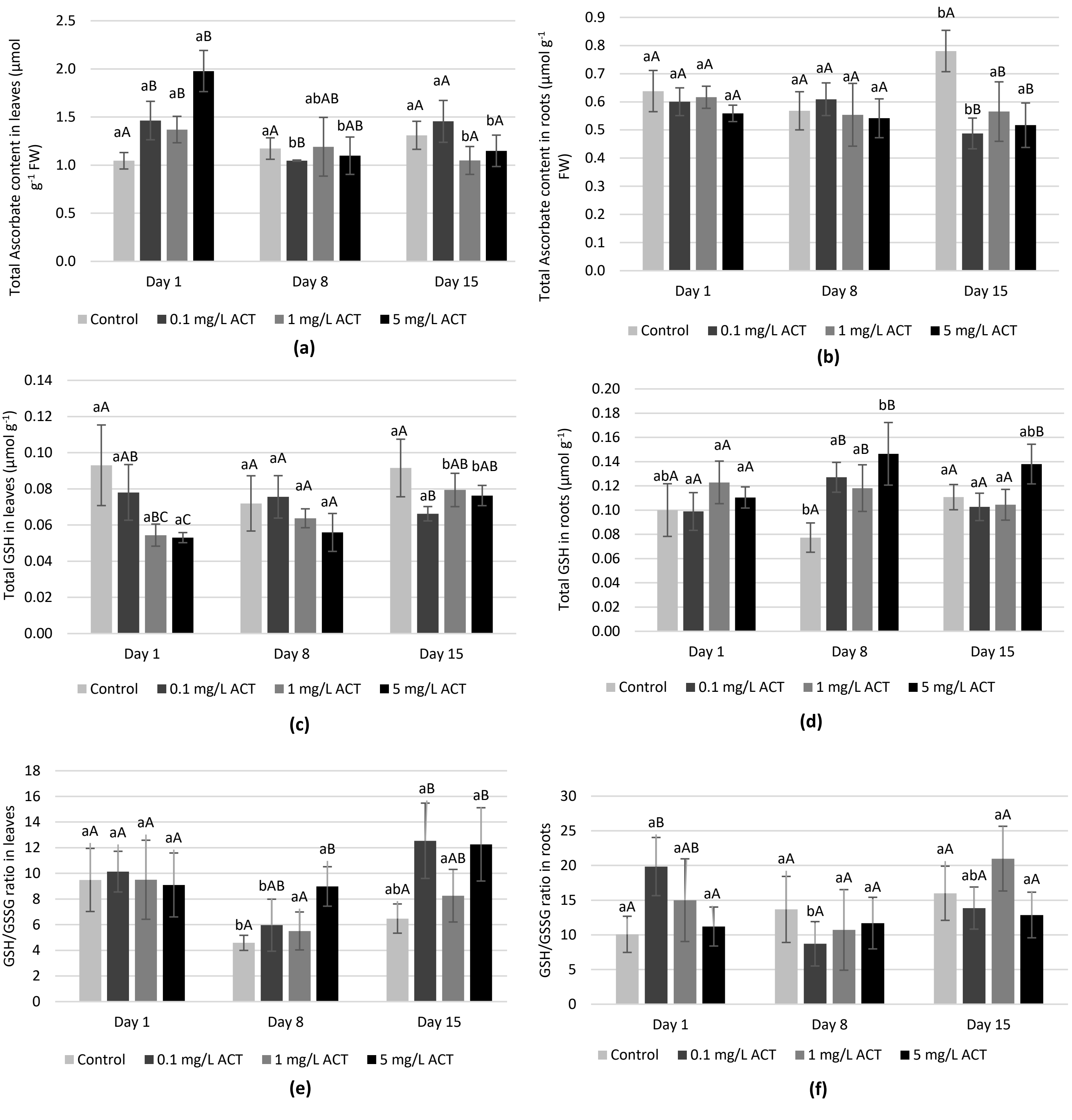

FIGURE S3 – Total Ascorbate content (µmol g^{-1} FW) in leaves (a) and in roots (b), Total GSH content (µmol g^{-1} FW) in leaves (c) and roots (d) and GSH/GSSG ratio in leaves (e) and roots (f) of *Lactuca sativa* under contamination with ACT for up to 15 days. Different uppercase letters represent significant differences between different ACT concentrations at the same timepoint and different lowercase letters represent significant differences between treatment duration for the same ACT concentration.
